# Metastatic signet ring cell adenocarcinoma of bone marrow with bilateral ovarian masses: a case report

**DOI:** 10.1186/1757-1626-1-332

**Published:** 2008-11-19

**Authors:** Deepali Jain, Nidhi Mahajan, Tejinder Singh

**Affiliations:** 1 Department of Pathology, Maulana Azad Medical College, New Delhi, India

## Abstract

We present a case of metastatic signet ring cell adenocarcinoma of bone marrow with radiologically proven bilateral ovarian masses in a 50 year old Asian Indian female. Even after thorough search no extraovarian primary site could be found. Based on overall clinicopathologic correlation, a diagnosis of metastatic signet ring cell adenocarcinoma of bone marrow with uncertain primary was established.

## Background

Marrow metastases in nonhematologic tumors are reported in virtually all types of malignancies. Tumors that commonly metastasize to the bone marrow are carcinomas arising in the prostate, breast, lung, and neuroblastoma [1]. Rarely epithelial tumors of the ovary also metastasize to the bone and bone marrow [2]. There are only few case reports in the literature describing bone marrow metastasis from primary as well as secondary krukenberg tumors of the ovary (Table 1) [3-8]. Herein we describe a case of bilateral ovarian masses, histopathology of which could not be done due to early demise of the patient, with metastatic signet ring cell carcinoma in the bone marrow. Even after thorough investigations a primary site of malignancy could not be detected. Based on all the findings a possibility of metastatic signet ring cell carcinoma of bone marrow and Krukenberg tumor with uncertain primary was kept.

**Table 1:** Previously reported patients of Krukenberg carcinoma with bone and marrow metastases

Author	year	Age/sex	Site of metastasis	Type of Krukenberg tumor	Outcome
Simecek A [3]	1937	NA	NA	Secondary	NA
Lowman and Kushlan [4]	1945	20/F	Vertebrae	Secondary	Died
Zeigerman JH [5]	1948	27/F	Osteoplastic metastases to spine, pelvis, ribs, clavicle, scapulae and upper humeri	Primary	Died
Engeler et al [6]	1976	Pregnant female	Osteoplastic metastases and necrosis of the marrow.	Primary	NA
Joshi VV [7]	1968	33/F	Ribs, pelvis, spine and greater trochanter of left Femur along with marrow dissemination.	Primary	Died
Metz et al [8]	1980 (Two cases)	1. 40/F 2. 29/F	1. Skull, spine, ribs and hip with marrow involvement 2. Skull and spine	Secondary	Died
Present case	2007	50/F	Osteolytic lumbar vertebrae with bone marrow involvement	Possibly secondary	Died

## Case presentation

**Figure 1:**
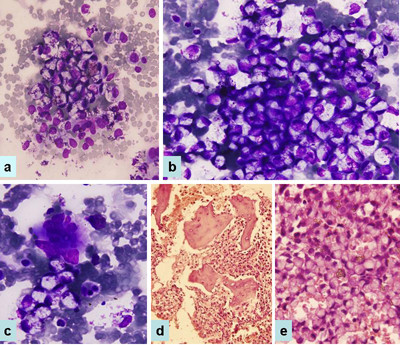
**Photomicrograph shows bone marrow aspirate smears (a, b, c) and trephine biopsy sections (d, e).** Total replacement of marrow elements by signet ring cells, a×40; b, c × 600, Wright Giemsa; d×40; e × 400, Hematoxylin and eosin.

The patient was a 50-year-old post menopausal Asian Indian lady, who had been in excellent health until approximately 7 days prior to admission, when she noticed the onset of lower abdominal discomfort and fullness. She admitted to a slight increase in lower abdominal girth however denied any gastrointestinal symptoms. Physical examination was essentially normal except for the presence of moderate pallor and slightly tender midline pelvic mass. Magnetic resonance imaging (MRI) revealed a large complex heterogeneously enhancing predominantly solid abdominopelvic mass in the midline extending towards left side. Bilateral ovaries were not visualized separately. MRI was suggestive of bilateral malignant ovarian masses. Ascitic fluid was present. There was an evidence of splenomegaly, approximately 18 cm in size. However pancreas and bilateral kidneys were normal in size and signal intensity pattern. Chest x-ray showed clear lung fields with hilar lymphadenopathy and mild cardiomegaly. X-ray of spine revealed lytic lesions in L4 and L5 vertebrae. Upper GI endoscopy was a normal study; however colonoscopy revealed grade II internal hemorrhoids. Cytologic examination of ascitic fluid did not show any malignant cells. As a part of metastatic work up mammography was also performed, that too was with in normal limits. Serum chemistries including liver and kidney function tests were normal except for the mildly elevated total bilirubin, urea and creatinine. Hematologic examination showed presence of bicytopenia, her hemogram findings were as follows: Hb 4.2 gm%, TLC 9,600 cumm, platelets 40,000 cumm. Peripheral blood smear exhibited leukoerythroblastosis. Bone marrow aspirate and biopsy revealed metastatic signet ring cell adenocarcinoma with near total replacement of normal bone marrow elements. Aspirate smears were hypercellular and replaced by large oval to polygonal cells having significant nuclear pleomorphism. The nuclei for the most part were eccentrically located. The cytoplasm varied from being finely vacuolated to possessing a single, large, clear vacuole (signet ring cells) (Figure 1). The cells were arranged individually or in small aggregates. Mucin stain showed strong cytoplasmic positivity. Bone marrow biopsy (Figure 1) revealed the intertrabecular spaces to be packed by similar malignant cells. There was almost complete replacement of normal hematopoeitic elements with increase osteoblastic and osteoclastic activity. The mucicarmine stain was positive. Based on all these findings a diagnosis of metastatic signet ring cell adenocarcinoma was established and advised to search for the primary site. No primary site could be found except for the presence of bilateral ovarian masses. The patient continued to deteriorate despite repeated blood transfusions and she died around 10 days after her admission. Post mortem examination could not be performed.

## Discussion

From review of clinical and autopsy material, the most common occurrence of marrow metastases in nonhematologic malignancies is in patients with carcinomas arising in the prostate, breast, lung, and neuroblastoma [1]. Epithelial tumors of the ovary especially mucinous carcinomas rarely metastasize to the marrow [2]. In the present case we found radiologically proven bilateral ovarian masses with signet ring cell adenocarcinoma in the bone marrow. Unfortunately, due to disseminated disease and early demise of the patient histopathology of ovarian mass could not be done. Autopsy was also not obtained. Even after extensive investigations including endoscopic examination of GIT, mammography and cervical biopsy, we were unable to find any extraovarian lesion. Therefore it was assumed that bone marrow metastasis would have been occurred either due to occult primary site in the GIT or to poorly differentiated mucinous carcinoma or primary Krukenberg tumor of the ovary. Poorly differentiated mucinous carcinomas show foci of typical signet ring cells, and the lesion may in parts resemble a Krukenberg tumor. However in the bone marrow we observed diffuse presence of signet ring cells. Diagnosis of primary krukenberg tumor requires survival for 5 years or longer or the presence of a detailed autopsy examination to rule out any other primary site. Both of these criteria were not met in our case, thus excluding primary Krukenberg tumor. Although histologic examination of ovarian masses could not be performed, radiologic features were suggestive of Krukenberg tumor. With the features described in this case, the ovaries are of metastatic origin until proven otherwise. It is not uncommon for a secondary Krukenberg tumor of the ovary to present first before the true extra-ovarian primary tumor is discovered. The majority of those are of gastric origin. Sometimes, this can require an exhaustive search. All tumors with microscopic features of the Krukenberg tumor in the ovary are metastatic, very rare examples may be primary. Joshi [7] accepted as primary 18 reported cases, including 11 in which autopsy examination revealed no evidence of an extraovarian source, and 7 in which the patient survived for 5 to 13 years after the removal of the ovarian tumor. To the best of our knowledge only few cases of Krukenberg carcinoma with bone and bone marrow metastases have been described in the literature. Zeigerman [5] described a case of primary Krukenberg tumor with osteoplastic metastases predominantly to axial skeleton. Joshi [7] reported a 33 year old Negro female with right ovarian tumor and metastatic lesions in the spine and greater trochanter of the femur. Even after thorough post mortem examination he could not find any extraovarian primary site; therefore considered it as a primary Krukenberg carcinoma. Subsequently Engeler et al [6] reviewed primary Krukenberg tumours in pregnancy and they found one patient with osteoplastic metastases and necrosis of the marrow. In 1980 Metz et al [8] described two patients of secondary Krukenberg carcinoma with bone marrow metastases. In both the cases primary tumor was present in gastric antrum. All the previously reported patients died due to disseminated disease, as was seen in the index patient. It seems likely that impairment of marrow function by tumor contributed significantly to the rapid deterioration of these patients. As seen in the present case and from review, the disease is a virulent one. The presence of ascitis is an ominous sign. The prognosis of primary Krukenberg carcinoma is less bleak than that of secondary Krukenberg tumor [7].

## Conclusion

The present case adds to the literature a rare case of metastatic signet ring carcinoma of marrow and possibly secondary Krukenberg carcinoma of the ovary with an uncertain primary site.

## Consent

A fully informed written consent was obtained from the patient family for the publication of this case report and accompanying images. A copy of the written consent is available for review by the Editor-in-Chief of this journal.

## Competing interests

The authors declare that they have no competing interests.

## Authors' contributions

DJ, NM, TS have made significant contributions by making diagnosis and intellectual input in the case and writing the manuscript.
